# Diagnostic yield of real-time PCR vs. NGS in pediatric FMF: insights from a Turkish cohort

**DOI:** 10.1515/med-2026-1451

**Published:** 2026-06-18

**Authors:** Emine Göktaş, Tuğba Deniz Kurnaz Demir, Ayşe Gül Zamani, Mahmut Selman Yildirim

**Affiliations:** Department of Medical Genetics, Faculty of Medicine, Necmettin Erbakan University, Konya, Türkiye

**Keywords:** autoinflammatory diseases, Familial Mediterranean Fever, *MEFV*, next-generation sequencing, real-time PCR

## Abstract

**Objectives:**

Familial Mediterranean Fever (FMF) is the most common monogenic autoinflammatory disease, especially in Mediterranean populations. This study compares the diagnostic value of real-time PCR and next-generation sequencing (NGS) in a Turkish pediatric cohort and assesses the benefits of comprehensive genetic panels.

**Methods:**

A total of 478 children with suspected FMF, seen between January 2022 and July 2024, were retrospectively analyzed. All underwent initial *MEFV* mutation screening via real-time PCR. Patients with negative or inconclusive results (n=39) were further tested using targeted NGS, covering *MEFV* and other autoinflammatory genes.

**Results:**

Real-time PCR detected at least one *MEFV* variant in 211 patients (44.1 %). The most common variants were M694V (41.6 %), E148Q (24.4 %), V726A (12.4 %), and M680I (10.9 %). Among the 39 patients further evaluated with NGS, some had additional or rare variants in *MEFV* or other genes such as *MVK, NOD2, STAT3*, and *NLRP12*, providing additional genetic findings, particularly in PCR-negative cases.

**Conclusions:**

Real-time PCR is effective for initial *MEFV* screening. However, NGS enhances diagnostic accuracy by identifying rare or complex variants, supporting its use in unresolved or atypical FMF cases.

## Introduction

Familial Mediterranean Fever (FMF) is the most common monogenic autoinflammatory disease, predominantly affecting populations of Mediterranean descent, including individuals of Arab, Armenian, Turkish, and Jewish heritage. It is characterized by recurrent episodes of fever, abdominal pain, and joint manifestations, often accompanied by peritonitis, pleuritis, and erysipelas-like skin lesions [1]. FMF is typically inherited in an autosomal recessive manner (OMIM: 249100), though rare autosomal dominant cases with variable penetrance have been reported (OMIM: 134610) [2]. It is caused by variants in the *MEFV* gene on chromosome 16p13.3, which encodes marenostrin/pyrin, a key regulator of the inflammasome and innate immunity [3]. Changes in *MEFV* can result in dysregulated inflammation through excessive production of interleukin-1 (IL-1), leading to the clinical manifestations observed in FMF [4].

The *MEFV* gene comprises 10 exons, with the majority of variants localized in exons 2 and 10. The *Infevers* database lists over 400 *MEFV* variants, categorized as benign, likely benign, pathogenic, likely pathogenic, or of uncertain significance (VUS) [1], 5]. Four common pathogenic exon 10 variants, M694V, M680I, V726A, and M694I, are prevalent across ethnic groups, while E148Q, in exon 2, is frequent in some populations but remains of uncertain pathogenicity [6], 7]. In regions with a high prevalence of FMF, these five founder mutations account for approximately 74 % of reported cases. While variants may also occur in other exons of the *MEFV* gene, their clinical significance can vary [8]. The genotype–phenotype correlation in FMF is complicated by regulatory genes (e.g., SAA and MICA), genetic heterogeneity, environmental factors, complex alleles, and *MEFV* variants associated with other diseases [9]. Elucidating the molecular basis of FMF is essential for accurate diagnosis and effective treatment. In the diagnostic process, variant-targeted methods such as real-time PCR, Sanger sequencing, pyrosequencing, strip assay, and the ARMS technique can be employed. However, next-generation sequencing (NGS) allows for comprehensive analysis by enabling the sequencing of the entire gene, as well as the evaluation of additional genes associated with disorders considered in the differential diagnosis [1], 10].

In Türkiye, the genetic diagnosis of pediatric FMF is traditionally managed via RT-PCR-based methods targeting common *MEFV* variants, primarily due to high disease prevalence and cost-effectiveness [1], 11]. However, a significant debate persists in the literature regarding the optimal diagnostic strategy. On one hand, Sözeri et al. [11] argued in a large cohort of 1,557 patients that short exon screening of the most frequent variants is sufficient for most cases, offering a 4.2-fold cost advantage over comprehensive sequencing [11]. Conversely, Kırnaz et al. [12] emphasized the superiority of NGS in a series of 3,230 patients, identifying rare variants and complex genotypes in 12.9 % of cases that targeted tests would have missed [12]. Supporting this, Tokgün et al. [13] demonstrated that NGS identified rare variants in 41 patients whose initial PCR results were negative, clearly illustrating how NGS can overcome the diagnostic limitations of PCR-based screening [13].

Despite these findings, current diagnostic strategies in Türkiye can be broadly categorized into (i) targeted mutation screening focusing on common MEFV variants and (ii) comprehensive sequencing approaches such as NGS, with no clear consensus on their optimal integration in pediatric clinical practice. This lack of standardization may lead to missed diagnoses in atypical or mutation-negative patients, as well as unnecessary use of advanced sequencing in clinically typical cases, thereby creating both diagnostic uncertainty and increased healthcare costs. Therefore, there remains a critical need for real-world data evaluating step-wise diagnostic strategies that balance cost-effectiveness with diagnostic accuracy, particularly in pediatric populations where phenotypic variability may further complicate clinical decision-making. In this context, the present study aims to assess the utility of integrating NGS following initial PCR-based screening and to propose a clinically applicable, step-wise diagnostic approach for pediatric FMF patients in Türkiye, with a particular focus on improving diagnostic yield in atypical cases.

## Materials and methods

### Sample collection and laboratory workflow

#### DNA extraction

DNA was extracted from EDTA-collected peripheral blood samples using TANBead^®^ (Taiwan Advanced Nanotech) or High Pure PCR Template kits (Roche Diagnostics) according to manufacturers’ instructions. DNA quality was assessed via NanoDrop™ (Thermo Fisher Scientific, Waltham, MA, USA), spectrophotometry, and stored at −20 °C.

#### Real-time PCR

Common *MEFV* variants were evaluated using the FMF-20 Multiplex Real-Time PCR Kit (SNP Biotechnology, Turkey) on a CFX96™ Real-Time PCR System (Bio-Rad Laboratories, Hercules, CA, USA), following recommended protocols. The SNP Biotechnology FMF Multiplex Real-Time PCR Kit (Cat. No: 11R-20-20) analyzes 20 *MEFV* mutations located in exons 1, 2, 3, 5, and 10, including E84K (exon 1); L110P, E148Q, E148V, E167D, E230K/Q, T267I, P283L, and G304R (exon 2); P369S (exon 3); F479L (exon 5); and M680I (G/C-A), M694I, M694V, K695R, V726A, A744S, and R761H (exon 10). While a direct comparison with Sanger sequencing was not performed, concordance between real-time PCR and NGS results for the variants included in the kit was observed in patients who underwent both methods, supporting the overall reliability of the assay.

#### Next-generation sequencing

Targeted sequencing was performed using a custom-designed gene panel (Roche Sequencing Solutions, Pleasanton, CA, USA) covering *MEFV* and other autoinflammatory-related genes (*ELANE, EPCAM, IL10, IL1RN, IL6, LPIN2, MVK, NLRP3, NOD2, PSTPIP1, TNFRSF1A,* and *TTR*). Library preparation and target enrichment were carried out according to the manufacturer’s protocol, including DNA fragmentation, adapter ligation, and PCR amplification, followed by hybridization-based capture of target regions. Sequencing was conducted on the DNBSEQ-G400™ platform (MGI Tech Co., Ltd., Shenzhen, China) using a paired-end sequencing mode (PE150). A minimum sequencing depth of 30× was achieved, with a mapping quality of 60 and a base quality score of Q≥30, ensuring ≥95 % coverage across all target regions. Variant calling thresholds were applied consistently across all samples, with a minimum variant allele fraction (VAF) of 20 % for heterozygous calls. Raw sequencing data were processed using the Genomize bioinformatics platform. The analysis pipeline included adapter trimming and alignment to the human reference genome (GRCh38/hg38) using the Burrows–Wheeler Aligner (BWA). Variant calling was performed using an optimized high-accuracy variant identification pipeline implemented within the Genomize platform. Identified variants were annotated using multiple databases, including ClinVar, gnomAD, and dbSNP. Functional annotation and variant effect prediction were performed using the Ensembl Variant Effect Predictor (VEP), together with in silico prediction tools integrated within the Genomize system. Variant interpretation and classification were performed according to the American College of Medical Genetics and Genomics (ACMG) guidelines. ACMG criteria were applied using a combined approach involving automated annotation within the Genomize platform and subsequent manual expert review. The classification process incorporated population frequency data, computational predictions, previously reported clinical significance, segregation data (when available), and relevant literature evidence. No standalone automated classification tool (e.g., InterVar) was used; instead, final variant classification was determined through expert evaluation in accordance with ACMG standards. All novel or clinically significant *MEFV* variants were confirmed by bidirectional Sanger sequencing.

**Figure 1:**
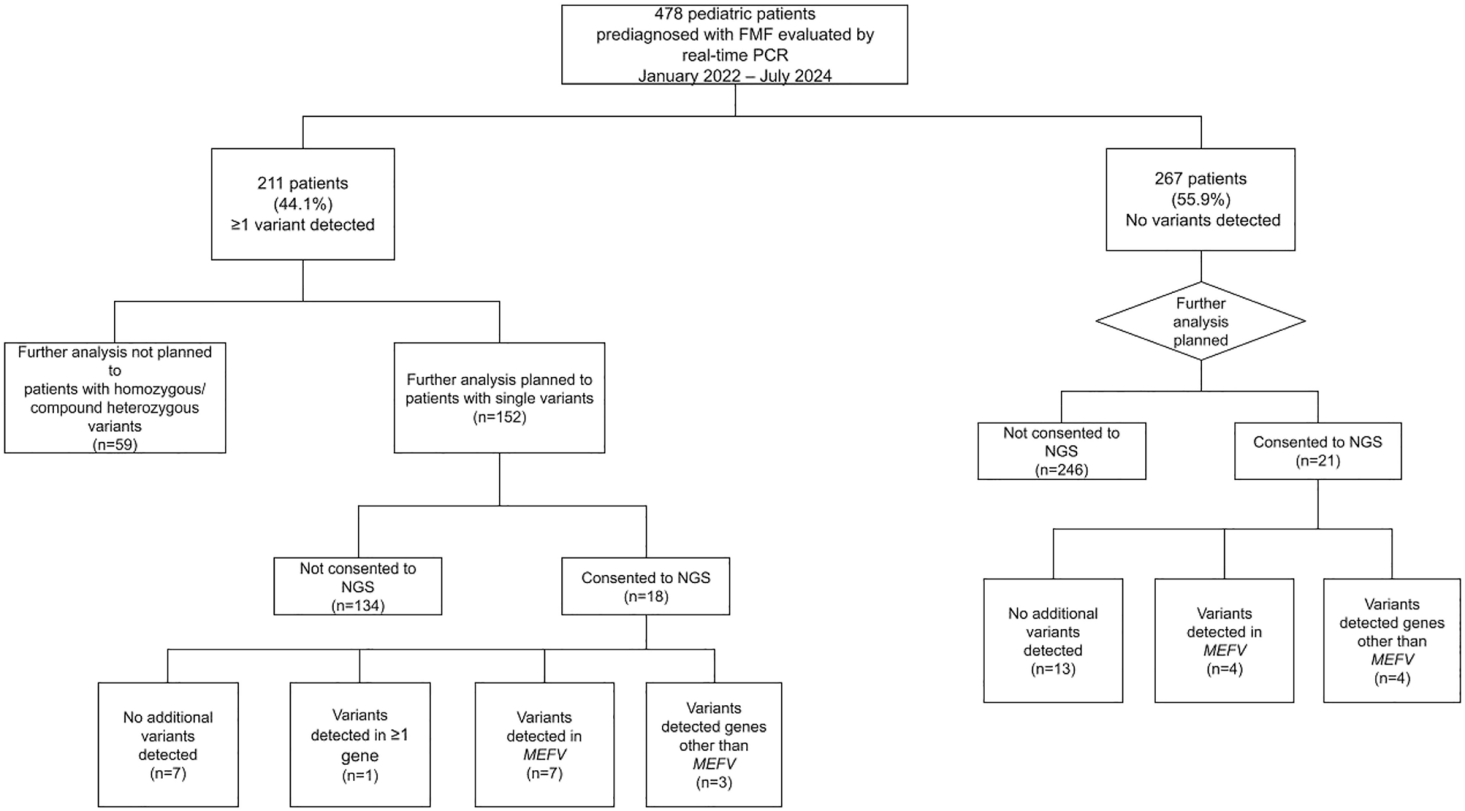
Flowchart of the stepwise genetic diagnostic algorithm applied to 478 pediatric patients with clinical suspicion of FMF.

### Statistical analysis

All statistical analyses were performed using IBM SPSS Statistics v29.0.2.0 (IBM Corp., Armonk, N.Y., USA). Descriptive statistics were expressed as frequencies and percentages. To compare the diagnostic yield of real-time PCR and NGS in the subset of 39 patients who underwent both methods, a paired analysis was performed using the McNemar test. A positive diagnostic outcome was defined as the detection of any clinically relevant variant regardless of ACMG classification by the respective method. For real-time PCR, this was limited to *MEFV* gene variants, whereas for NGS, this encompassed all 13 genes included in the targeted autoinflammatory panel. Given the small number of discordant pairs observed, the exact McNemar test (two-sided binomial test) was used instead of the asymptotic chi-square approximation. A p-value of less than 0.05 was considered statistically significant.

#### Ethical statement

This study was approved by the Local Ethics Committee of Necmettin Erbakan University for Clinical Studies (approval date and number: 2024/5305) and was conducted in accordance with the Declaration of Helsinki. 

#### Informed consent

Written informed consent was obtained from all legal guardians.

## Results

### Analysis of *MEFV* variants in FMF-suspected patients

A total of 478 pediatric patients with suspected FMF were analyzed (mean age: 8.04 years; 51.1 % female). Initial screening with real-time PCR targeting 20 common *MEFV* mutations identified at least one variant in 211 patients (44.1 %), while 267 (55.9 %) showed no detectable change. Due to unresolved clinical suspicion, 39 patients (21 PCR-negative, 18 PCR-positive) underwent additional targeted NGS. In this subgroup, all PCR-positive cases were confirmed by NGS, while 8 of 21 PCR-negative patients were reclassified as positive and 13 remained negative; no PCR-positive/NGS-negative cases occurred. Figure 1 The exact McNemar test demonstrated a significant advantage for NGS (p=0.008). When restricted to *MEFV* variants, four discordant pairs were identified without statistical significance (p=0.125), whereas inclusion of all autoinflammatory genes increased discordant pairs to eight, restoring significance (p=0.008). These findings suggest that the higher diagnostic yield of NGS may reflect broader gene coverage, although results should be interpreted cautiously given the small sample size.

### Real-time PCR findings

**Figure 2:**
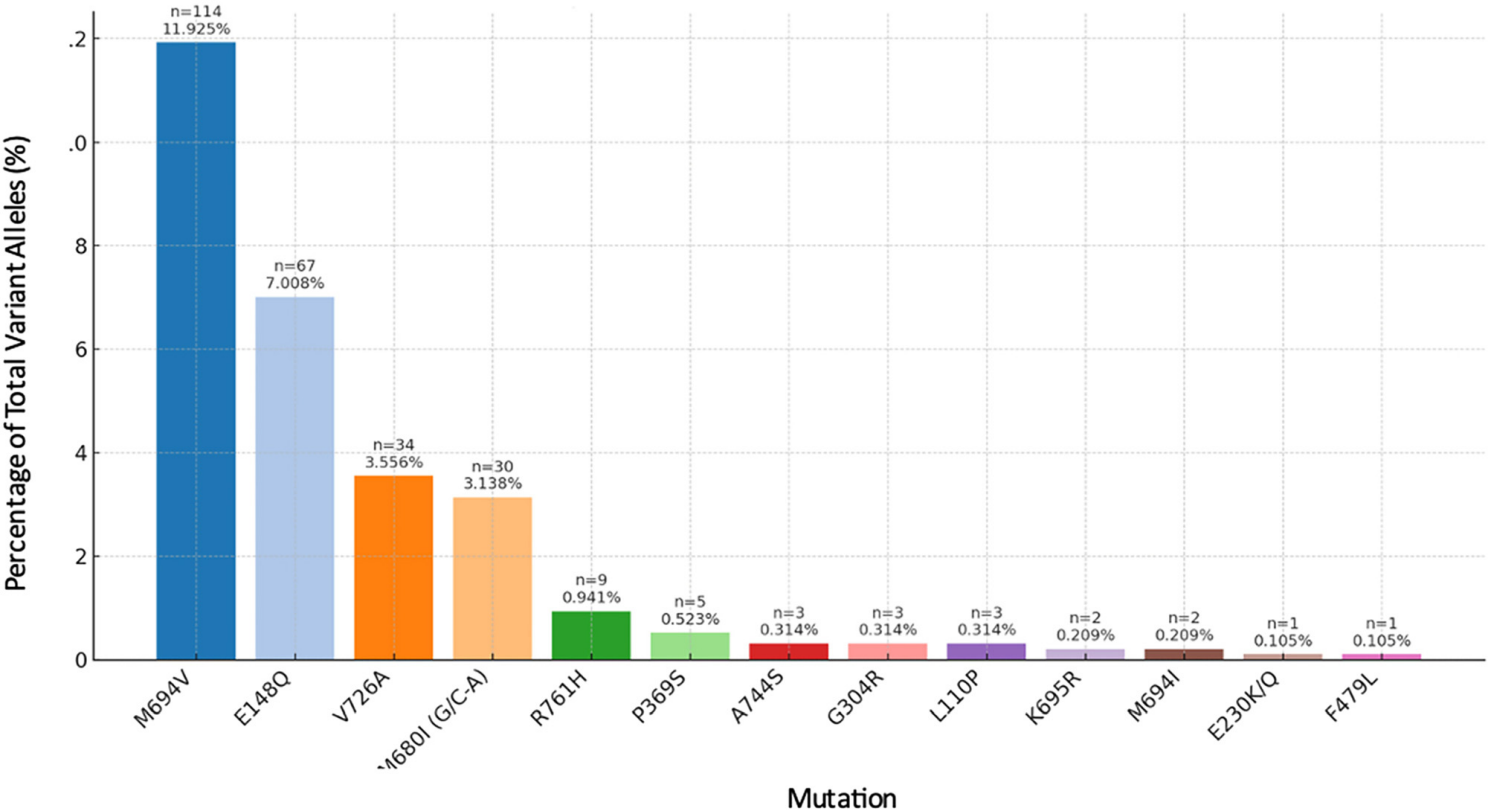
Percentage of total variant alleles and distribution of *MEFV* variants identified in the study population.

Among the 211 PCR-positive patients, 179 carried a single *MEFV* variant, and 32 harbored complex alleles. A total of 274 variant alleles were detected. The most frequent variants were M694V (41.6 %; VAF: 11.9 %), E148Q (24.4 %; VAF: 7.0 %), V726A (12.4 %; VAF: 3.6 %), and M680I (10.9 %; VAF: 3.1 %). Less common variants included R761H, P369S, A744S, and G304R. Two types of variants were detected in 30 patients (14.2 % of PCR-positive cases). Three distinct variants, though less frequent, were identified in a few patients. The majority of variants were heterozygous; homozygous forms were particularly observed in M694V and E148Q. Figure 2 illustrates the distribution of the *MEFV* variants and their respective frequencies.

### NGS findings

Among the 39 patients who underwent NGS, 19 (48.7 %) had additional genetic findings that were not identified by real-time PCR. Among the patients in whom PCR previously detected a single *MEFV* variant, 7 were found to have additional *MEFV* variants, and 3 carried variants in other autoinflammatory genes (*NLRP12, ELANE,* and *TNFRSF1A*). Additionally, one patient in this group harbored two distinct variants: one in *MEFV* and another in *NLRP12.* In the PCR-negative subgroup, NGS identified *MEFV* variants in 4 patients, including one case with a novel homozygous p.S575L variant, while an additional 4 patients had variants in non-*MEFV* autoinflammatory genes (*NOD2, STAT3*, and *MVK*) (Table 1). These findings demonstrate that NGS provides added diagnostic value in both PCR-positive and PCR-negative patients by identifying rare, non-hotspot, or complex genotypes that may be missed by targeted assays.

**Table 1:** Comparison of real-time PCR and NGS results in patients with suspected FMF.

Patient no	PCR status	Real-time PCR (MEFV)	NGS (MEFV)	Concordance	Additional NGS findings (non-MEFV)	ACMG classification^a^
1	Positive	V726A/0	V726A/0	Yes	NLRP12 L748V	VUS
2	Positive	V726A/0	V726A/0	Yes	–	–
3	Positive	M694V/0	M694V/R202Q	Partial	–	B
4	Positive	M694V/0	M694V/R202Q	Partial	–	B
5	Positive	M694V/0	M694V/R202Q	Partial	–	B
6	Positive	M694V/0	M694V/R202Q	Partial	–	B
7	Positive	M694V/0	M694V/R202Q	Partial	NLRP12 G52S	VUS
8	Positive	M694V/0	M694V/R202Q	Partial	–	B
9	Positive	M694V/0	M694V/R202Q	Partial	–	B
10	Positive	M694V/0	M694V/0	Yes	–	P
11	Positive	M694V/0	M694V/0	Yes	–	P
12	Positive	E148Q/0	E148Q/0	Yes	TNFRSF1A R121Q	VUS
13	Positive	E148Q/0	E148Q/R202Q	Partial	–	VUS
14	Positive	E148Q/0	E148Q/0	Yes	–	VUS
15	Positive	E148Q/0	E148Q/0	Yes	–	VUS
16	Positive	E148Q/0	E148Q/0	Yes	–	VUS
17	Positive	E148Q/0	E148Q/0	Yes	–	VUS
18	Positive	M680I/0	M680I/0	Yes	ELANE V112^a^	LP
22	Negative	–	S575L/S575L	–	–	VUS
26	Negative	–	R202Q/0	–	–	B
28	Negative	–	–	–	MVK V377I	P
30	Negative	–	–	–	NOD2 R703C	LB
31	Negative	–	E225D/0	–	–	VUS
36	Negative	–	R202Q/0	–	–	B
37	Negative	–	–	–	NOD2 R412C	VUS
38	Negative	–	–	–	STAT3 S372F	VUS
39	Negative	–	–	–	–	–

P, pathogenic; LP, likely pathogenic; B, benign; LB, likely benign; VUS, variant of uncertain significance. ^a^ACMG, classification of the variant detected by NGS.

## Discussion

In the present study, we found that real-time PCR alone detected at least one *MEFV* gene variant in 211 out of 478 pediatric patients with suspected FMF (44.1 %), underscoring its substantial diagnostic contribution as a first-line approach. The integration of NGS resulted in a modest increase in detection rate to 44.9 % (n=215), indicating a limited but measurable incremental diagnostic yield, largely driven by the detection of additional variants of uncertain or limited clinical significance. In addition, NGS enabled the identification of variants in genes associated with other autoinflammatory disorders in a small subset of patients. Taken together, these findings support the utility of a tiered diagnostic strategy, in which real-time PCR serves as an initial screening tool, while NGS is reserved for selected cases with unresolved or atypical clinical presentations. To the best of our knowledge, this is among the few studies to directly compare these two diagnostic approaches in a large pediatric cohort from Türkiye, expanding the current understanding of the *MEFV* mutation spectrum and emphasizing the importance of a balanced, context-specific genetic testing strategy in regions with high FMF prevalence. It should be noted that NGS was not performed in all eligible patients but only in a subset who consented to further testing. Therefore, the incremental diagnostic yield observed in this study likely underestimates the true diagnostic contribution of NGS in an unselected population.

In our cohort, *M694V* was the most frequently detected variant, accounting for 41.6 % of all alleles, followed by *E148Q* (24.4 %), *V726A* (12.4 %), and *M680I* (10.9 %). Other less frequent variants, including R761H, P369S, G304R, and L110P, were observed at low frequencies, consistent with previous Turkish cohorts, where these variants similarly appear sporadically and contribute to the overall genetic heterogeneity of FMF [12], 13]. However, the novel p.S575L variant identified in our cohort requires further functional and clinical validation before any definitive conclusions regarding its pathogenicity can be drawn. These findings are consistent with previous large-scale studies from Türkiye, including one cohort of 3,167 patients in which 46.7 % carried at least one *MEFV* variant, with *M694V* and *V726A* predominating, followed by *E148Q* and *M694I* [14], and another study of 3,230 individuals reporting *R202Q*, *E148Q*, and *M694V* as the most frequent variants [12]. Similarly, regional pediatric data from Egypt and other Mediterranean populations also demonstrate the predominance of *M694V* – 50.5 % in a cohort of 500 patients from Cairo [15] and 42.8 % in another regional study, followed by *M680I* and *E148Q* [11]. Despite overall concordance, the relative frequencies of *E148Q*, *V726A*, and less common variants exhibit variability across studies, likely reflecting differences in geographic subregions, population structure, referral patterns, patient selection criteria, and methodological approaches, including targeted PCR vs. NGS-based detection [11], [12], [13], [14], [15]. These comparisons highlight both the consistency of M694V as the dominant pathogenic allele across Turkish and Mediterranean pediatric populations and the genetic heterogeneity of FMF, underscoring the importance of population-specific mutation profiling to guide accurate and efficient diagnostic strategies.

Among the additional variants detected by NGS, R202Q emerged as the most frequent, most often in combination with M694V (7 of 8 cases). Although R202Q is generally classified as a benign or likely benign variant according to ACMG criteria, its clinical significance remains controversial. Previous studies have suggested that the M694V/R202Q compound heterozygous genotype may be associated with more severe clinical manifestations, including higher rates of arthritis and increased disease severity scores [16]. In addition, emerging functional data indicate that carriers of the R202Q variant may exhibit altered inflammatory responses, despite no direct effect on pyrin function [17]. Taken together, these findings suggest that R202Q may have a potential modifier role rather than a direct pathogenic effect, particularly in combination with disease-causing variants such as M694V. Therefore, while its routine inclusion in screening panels remains debatable, consideration of R202Q may be informative in selected clinical contexts, and further large-scale and functional studies are warranted. Complex genotypes, including compound heterozygous or triple variant combinations, were observed in 15.2 % of PCR-positive patients, comparable to previous reports from Türkiye, where the frequency was 12.9 % [12], underscoring the challenges in correlating genotype and phenotype in FMF and the added value of comprehensive NGS analysis for precise diagnosis.

In addition to detecting *MEFV* variants, NGS revealed co-existing variants in genes associated with other systemic autoinflammatory diseases (SAIDs), including *MVK*, *NOD2*, *STAT3*, and *TNFRSF1A*, which are linked to distinct clinical entities such as HIDS, Crohn-like inflammatory conditions, and TRAPS, all of which may present with recurrent fever episodes similar to FMF and therefore complicate the differential diagnosis [18]. Among the four variants identified in our cohort, only one was classified as pathogenic, two were VUS, and one was likely benign, indicating that non-*MEFV* variants should be interpreted cautiously, particularly in pediatric patients with overlapping or atypical phenotypes. Emerging tools such as large language models may further support the interpretation of complex genetic datasets by integrating large-scale genomic and literature-based evidence; however, their clinical applicability in this context still requires careful validation [19].

When comparing targeted mutation screening with more comprehensive sequencing approaches, our findings of a modest increase in variant detection with NGS are consistent with previous reports in the literature. In our cohort, the addition of NGS increased the detection rate from 44.1 to 44.9 %, indicating a limited but measurable contribution of extended analysis. Similarly, in a large Turkish cohort, targeted screening identified the majority of *MEFV* variants, while additional testing revealed extra mutations in only 4.3 % of patients [20]. Comparable findings were reported by Moradian et al., where sequence analysis identified additional variants in 4.3 % of heterozygous individuals not detected by initial screening [21]. In another study by Mattit et al., sequencing of exon 10 in patients with inconclusive initial results led to the detection of additional variants in 12 % of cases [22]. Similarly, a large-scale analysis by Sözeri et al. demonstrated that while 7.6 % of patients remained negative after targeted mutation screening, this proportion decreased to only 0.3 % following full-gene sequencing, indicating that comprehensive analysis may substantially reduce the number of genetically unresolved cases in selected cohorts [11]. These observations collectively suggest that, while additional variants can be detected beyond targeted panels, the overall increase in diagnostic yield is generally modest but may be clinically meaningful in a subset of patients.

From a cost perspective, this limited incremental yield should be carefully balanced against the substantially higher expense of comprehensive sequencing approaches. Sözeri et al. reported that extended genetic analysis may increase costs by approximately 4.2-fold compared to targeted testing [11]. In our cohort, sequencing of the entire *MEFV* gene was approximately 2.2 times more costly than targeted variant analysis, while the use of a broader NGS panel including other autoinflammatory disease-related genes increased costs up to 4.8-fold. Taken together, these findings suggest that targeted analysis of common *MEFV* mutations captures the majority of clinically relevant variants in high-prevalence populations, whereas more comprehensive approaches provide incremental diagnostic benefit at a significantly higher cost.

Therefore, rather than replacing targeted screening, extended sequencing approaches such as whole-gene analysis or NGS panels appear to function as complementary tools, particularly in selected clinical scenarios, including patients with PCR-negative results despite strong clinical suspicion, atypical or severe presentations, positive family history suggestive of complex inheritance, or cases in which a single variant identified by PCR does not fully explain the clinical phenotype. This supports a stepwise and cost-conscious diagnostic strategy that optimizes both clinical utility and resource utilization. Although specific *MEFV* or non-*MEFV* variants may theoretically influence disease severity or therapeutic decisions, this study did not include treatment data, and genotype-directed management was beyond the scope of our analysis.

### Study strengths and limitations

One of the main strengths of our study is the large cohort size, which allows for a comprehensive evaluation. The use of both real-time PCR and targeted NGS reflects a practical and stepwise diagnostic approach aligned with current clinical practice. However, a significant number of patients eligible for further genetic analysis did not participate in the NGS phase. This limited participation may have led to an underestimation of rare variants and represents a key limitation of the study. Another limitation of our study is the lack of a systematic demographic and clinical comparison between PCR-positive, PCR-negative, and NGS-tested groups, which may restrict the interpretability of diagnostic yield differences. Our study focuses exclusively on the genetic diagnostic workflow and does not provide longitudinal clinical or treatment data. Therefore, we cannot comment on genotype-specific management strategies or therapeutic outcomes. Future studies integrating genetic, clinical, and treatment data are warranted to explore potential genotype-phenotype-treatment correlations.

## Conclusions

In this study, we utilized both real-time PCR and NGS methods for the diagnosis of FMF. Real-time PCR remains a rapid and cost-effective approach for detecting common *MEFV* mutations. However, its capacity is limited in identifying rare, novel, or complex variants. In contrast, NGS enables a comprehensive analysis of the *MEFV* gene and additional genes involved in the differential diagnosis of autoinflammatory disorders. Our findings highlight that NGS should be considered a second-line, problem-solving tool rather than a universal first-line test.
